# Using Alcohol and Sleep Sensors to Understand Blackout Risk in Young Adults’ Natural Settings (The Lights Out Study): Protocol for an Intensive Longitudinal Pilot Study

**DOI:** 10.2196/83980

**Published:** 2026-01-21

**Authors:** Veronica L Richards, Ashlea Braun, Michael R Sladek, Junru Zhao, Thad Leffingwell, Sydney Newell Chesebro, Julie M Croff

**Affiliations:** 1 TSET Health Promotion Research Center, Stephenson Cancer Center, University of Oklahoma Health Sciences, 4502 E 41st St, Tulsa, OK, 74135, United States, 1 918-660-3176; 2 Department of Health Promotion Sciences, Hudson College of Public Health, University of Oklahoma Health Sciences, Tulsa, OK, United States; 3 Department of Psychology, The University of Oklahoma, Norman, OK, United States; 4 Department of Pediatrics, College of Medicine, University of Oklahoma Health Sciences, Oklahoma City, OK, United States; 5 Department of Psychology, Oklahoma State University, Stillwater, OK, United States; 6 Department of Rural Health, Oklahoma State University Center for Health Sciences, Tulsa, OK, United States

**Keywords:** alcohol-induced blackout, alcohol use, cannabis, college student, ecological momentary assessment, heavy drinking, naturalistic research, nicotine, polysubstance use, sleep, wearables, young adult

## Abstract

**Background:**

Alcohol-induced blackouts (AIBs) are a serious consequence of alcohol use that are strongly associated with experiencing excess alcohol−related harms. AIBs are common and recurrent among young adults who drink. The risk factors for AIBs include dynamics of alcohol use (quantity, speed, duration), alcohol-related behaviors (eg, playing drinking games, not using protective behavioral strategies), and factors related to the subjective experience of alcohol intoxication (eg, expectancies, motivations).

**Objective:**

This study seeks to examine 2 modifiable behaviors that have been shown to impact both alcohol consumption and subjective experiences of intoxication and may therefore be associated with AIB risk: (1) other substance use and (2) sleep.

**Methods:**

Approximately 50 participants will be recruited to participate in this study. Interested individuals will complete an online screening assessment, and those who are eligible (young adults who report recent heavy episodic drinking and AIBs) will be invited to an in-person baseline visit. At the baseline visit, participants will complete a baseline assessment, be fitted with a wrist-worn alcohol sensor (BACtrack Skyn) and a sleep or activity ring sensor (Oura ring), and receive training on the study protocol. Participants will complete a 14-day intensive data collection period consisting of twice daily scheduled mobile surveys and participant-initiated drinking surveys with hourly follow-ups. Participants will also wear the alcohol and sleep or activity sensors continuously during this 14-day period. After the intensive data collection period ends, participants will complete an in-person return visit to return their sensors, complete a follow-up survey, and receive compensation. The data will be processed and cleaned, and analyses will include multi-level structural equation models.

**Results:**

This study was funded in July 2025. Data collection is projected to span January 2026 through June 2026.

**Conclusions:**

This study seeks to understand 2 key modifiable behaviors that may be associated with increased AIB risk by leveraging multiple forms of innovative measurement. The integration of ecological momentary assessments with 2 sensors to capture alcohol use and sleep also supports potential applications in future digital interventions. This study will further enhance our preliminary data on the feasibility and acceptability of these methods, providing opportunities for conducting future research on a larger scale.

## Introduction

### Alcohol-Induced Blackouts

Alcohol-induced blackouts (AIBs) are a serious consequence of alcohol use characterized by anterograde amnesia while the individual is still awake and interacting with their environment and those around them [1]. AIBs are common and recurrent in young adult drinkers, with approximately 50% reporting past-month AIBs and about 80% experiencing at least one during their 4 years of college (for students) [2,3]. Individuals who engage in heavy drinking and report recent AIBs are a particularly “risky” group of young adults who experience AIBs at a rate of about 1 in every 3 drinking days [4]. The frequency with which AIBs occur is of concern given their strong association with experiencing increased numbers of alcohol-related consequences, acutely (eg, sexual violence) and prospectively (eg, alcohol use disorder) [5-12].

A number of risk factors for experiencing AIBs have been identified. The dynamics of alcohol consumption that serve as AIB risk factors include the quantity of alcohol consumed, speed at which intoxication occurs, and the amount of time spent becoming intoxicated [4,13-16]. Behaviors that impact alcohol dynamics have also been identified as AIB risk factors, including pregaming, participating in drinking games, using fewer protective behavioral strategies, and modifying diet to intensify or offset alcohol ingestion [17-21]. Additional evidence suggests that the subjective experience of intoxication, including alcohol use expectancies, daily motivations to use, and the experience of intoxication (ie, how intoxicated one feels), may also serve as important AIB risk factors [22-27]. This study seeks to examine 2 modifiable behaviors that have been demonstrated to impact both alcohol consumption and subjective experiences of intoxication: (1) other substance use and (2) sleep.

### Other Substance Use

Relative to single substance use, combined substance use has been associated with greater acute harms (eg, driving while intoxicated, overdose), chronic harms (eg, cancer), as well as heavier use [28-31]. About 20% of young adults who drink report combining alcohol with cannabis or nicotine [32]. The rates of cannabis and nicotine use among young adults continue to increase as markets for cannabis and nicotine (particularly e-cigarettes) rapidly evolve [33,34]. The effects of, and motives for, combined use vary by substance [35,36]. The effects of nicotine on subjective intoxication are mixed, but nicotine is consistently cited to offset the sedative effects of alcohol, allowing the individual to stay awake or out longer and drink more [31,37]. Cannabis is often combined with alcohol to enhance their effects [38] but may result in greater levels of cognitive impairment and impulsive decisions.

The effects of combined substance use on AIB risk have been mixed. Some studies report increased risk for AIBs when combining alcohol with cannabis [39,40] or nicotine [41], while others show no added risk associated with either [8,17,42]. In each of these studies, the timing of other substance use relative to alcohol use (eg, proximity to alcohol; order in which substances combined) may be a driving factor for such discrepancies. In the only study to consider same-day (ie, when alcohol and cannabis are used on the same day without overlapping effects) versus simultaneous alcohol-cannabis use (ie, when overlapping effects are experienced) on AIB risk, simultaneous use predicted AIB risk, but same-day use did not among college students [39].

### Sleep

Approximately 20%‐30% of young adults report recent sleep disturbances [43,44], and ~65% report past-month sleep problems [45,46]. Poor sleep has been associated with heavier drinking the next day (higher peak intoxication and higher drink count; ie, within-person) [47] and on average (ie, between-person) [46,48]. The relationship between alcohol quantity and alcohol-related consequences is stronger among individuals who report worse sleep or insomnia, on average [46,48-50]. Only 1 study to date has been published on the topic of sleep and AIB risk, which showed stronger associations between alcohol and AIBs among heavy drinking college students with more severe insomnia (compared to less severe insomnia) [49]. Poor sleep quality has further been associated and found to interact with greater endorsement of drinking motives, resulting in increased consequences [44]. This may be explained by research indicating that poor sleep may negatively impact cognitive functioning [51,52], decrease inhibition [53], and impair decision-making [54]. Most of these studies have been cross-sectional examinations of college students.

### Objectives

This study will expand on previous research investigating the associations between substance use, sleep, and AIBs in a sample of college and nonstudent young adults. The first aim is to examine the associations between combined alcohol and other substance use and AIBs. We hypothesize that simultaneous alcohol-cannabis use and simultaneous alcohol-nicotine use will be associated with increased odds of experiencing an AIB, compared to same-day (ie, alcohol and cannabis or nicotine use without overlap) and alcohol-only days. The second aim is to examine the associations between sleep (self-report quality and sensor-based measures; eg, rapid eye movement [REM] sleep) and AIBs. We hypothesize that days with worse past-night’s sleep (relative to an individual’s average sleep) will be associated with increased odds for AIBs. We will also explore the interaction between combined substance use and sleep on AIB risk via moderation.

## Methods

### Study Design

This is an intensive longitudinal study comprising an in-person baseline visit, 14-day intensive data collection period involving ecological momentary assessments (EMA) and sensor-wear regimen, and in-person return visit.

### In-Person Baseline Visit

Participants will receive a reminder email the day before their scheduled baseline visit and a text message reminder the morning of the visit. The in-person baseline visit is estimated to take approximately 1 hour to complete. Upon arrival, study staff will obtain informed consent. Participants will then complete a baseline survey on their demographics, recent alcohol and other substance use, alcohol-related consequences, sleep, and more (Table 1) via the Insight mHealth platform [55]. Following the completion of the baseline assessments, a trained study team member will provide an overview of study procedures and assist participants with setting up their devices. Participants will install 3 iOS apps on their personal iPhone (BACtrack Skyn, Oura, and Insight mHealth Platform). The study team member will assist the participant in selecting the correct sizes for each sensor (wrist and ring). The participant will be invited to join the “team” for each sensor’s research platform (Skyn, Oura) to share their data. They will connect each sensor to their phones via Bluetooth. The study team member will provide a tutorial regarding how to use each device and the survey platform, including the schedule of surveys. Before leaving the baseline visit, participants will also sign up for a time or date to return their sensors and receive payment within 2 business days (whenever possible) of the end of the data collection period (14 d).

**Table 1.** Summary of items assessed for the Lights Out study.

Domain (measures)	Baseline	Scheduled EMAs^a^	Drinking EMAs
Sociodemographics			
Age^b^, sex^b^, race, ethnicity, height, weight, sexual orientation	✓		
Living arrangement, relationship status, children, student status^b^, employment, income, social desirability	✓		
Proximity to study site^b^, willingness to wear sensors^b^	✓		
Alcohol use			
Family history of alcohol use disorder [56]	✓		
Age of alcohol use onset, past-year alcohol use disorder identification test [57], past 3-month typical alcohol use^b^ [57], past 3-month peak and duration, past 3-month alcohol-induced blackouts [49], past 3-month alcohol-related consequences [58]	✓		
Drink count^c^, start and stop time of drinking^c^, alcohol-induced blackouts^d^ [49], alcohol-related consequences^d^^e^ [58,59]		✓	✓^f^
Attitudes toward drinking [60]	✓		
Subjective intoxication^c^ [22]		✓	✓
Risky drinking behaviors (eg, using beer funnels, playing drinking games)^e^		✓	
Protective behavioral strategies^d^^e^ [61]		✓	
Situational drinking context (where and who drinking occurred with)		✓	✓
Willingness and expectancies to experience an alcohol-induced blackout^e^		✓	
Motives for drinking^e^ [58]^62^		✓	
Other substance use			
Past 3-month other substance use (cannabis, nicotine, unprescribed stimulant medication, other)	✓		
Current use of other substances (cannabis, nicotine, unprescribed stimulant medication, other), modes of nicotine and cannabis use^f^, timing of use relative to alcohol^e^		✓	✓
Cannabis-related consequences (adapted from Morin [63])		✓^e^	
Sleep			
Insomnia severity [63]	✓		
Sleep quality^f^ [64,65]	✓	✓	
Other measures			
Adverse childhood experiences [66]	✓		
Impulsivity [67]	✓		
Consumption of nonalcoholic beverages [68]		✓^g^	
Diet- and food-related behaviors^f^ [69-72]	✓	✓	✓
Mood and energy levels		✓	✓

a EMA: ecological momentary assessment.

b Assessed only on the screening survey.

c Only assessed in the evening EMA if morning EMA was skipped.

d Adapted for daily use.

e Not assessed on evening EMA.

f Past hour drink counts only.

g Not assessed on morning EMA.

### Intensive Data Collection Period

#### Overview

The intensive data collection period will begin the morning immediately following the baseline visit and will continue for 14 consecutive days. It will involve three primary components: (1) wearing the wrist-worn alcohol sensor, (2) wearing a finger-worn activity or sleep sensor, and (3) responding to brief surveys on a mobile device. Participants will be asked to wear the alcohol sensor and Oura ring continuously (including while asleep) except for while showering or charging the devices. Each day, participants will receive a reminder through the Insight mHealth Platform to sync their sensors with their respective apps. They will also receive a reminder every fourth day to charge their sensors. If technical issues arise with the sensors or apps, participants will be instructed to contact the study team. Participants will be instructed to continue to wear the devices experiencing the technical issue until they are able to meet with the study team. Technical issues will be addressed first internally and escalated to the respective company as needed. If the technical issue cannot be resolved immediately, devices will be exchanged for another device for the remainder of the study period. Broken devices will be exchanged as needed.

#### Alcohol Sensor

The alcohol sensor used in this study will be the BACtrack Skyn sensor. This sensor measures transdermal alcohol concentration (TAC) from the skin every 20 seconds, providing a (near) real-time, continuous, objective measure of alcohol intoxication. TAC is analogous and highly correlated with blood or breath alcohol concentration [73]. The sensors have a 10-day battery life and can be offline for extended periods without halting data collection or losing data.

#### Activity/Sleep Sensor

The Oura Ring will be used to assess sleep. Oura’s sleep tracking algorithms show good agreement with polysomnography, with no differences in sleep onset latency, total sleep time, or wake after sleep onset [74]. The ring has a 7-day battery life, can be offline for extended periods without halting data collection or losing data, and is sweatproof and waterproof.

#### Surveys

All surveys will be administered through the Insight mHealth platform. If Insight experiences any technical issues, surveys will also be programmed in REDCap (Research Electronic Data Capture) and delivered to participants via text message. Participants will be sent 2 surveys daily (see Table 1 for measures) corresponding to the morning and evening. An adaptive random prompting schedule will be programmed to each participant’s sleep-wake cycle. The morning survey (~5‐10 min) will open 30 minutes after typical wake-up time, and the evening survey (~3‐5 min) will open 7 hours after typical wake-up time. Each survey will be available for 3 hours, after which time the survey will be “locked” and considered missing. Participants will receive up to 2 reminders for each morning and evening survey at hourly intervals. Participants will also be asked to complete brief, self-initiated surveys when initiating alcohol use. Following the first drinking survey, participants will receive hourly follow-up drinking surveys. If the follow-up is not completed within 30 minutes, they will receive 2 additional reminders in 30-minute intervals. Follow-up surveys will end after the participant indicates that they are finished drinking, or they miss all prompts (initial follow-up prompt plus 2 reminders).

### In-Person Return Visit

At the end of the data collection period, participants will receive an email reminder the day before their scheduled return and a text message reminder the morning of their return. They will receive up to 3 reminders if they do not come in at their scheduled time or date. At the return visit, participants will return their sensors, complete a brief (~3 min) survey about their experience in the study, and receive compensation.

### Setting and Participants

Recruitment will occur in Tulsa, Oklahoma, and surrounding areas. We will post flyers at businesses in the area (eg, retail stores, restaurants, professional office buildings, community centers). We may also post advertisements on social media platforms (eg, Instagram, Facebook). Flyers and advertisements will include a study email address to contact for more information on participating as well as a link to the study website.

The inclusion criteria for this study include (1) age of 18‐25 years (college and nonstudent young adults); (2) report past 3-month behaviors that include drinking 4+/5+ drinks (women or men) at least once a week, on average; (3) report experiencing at least 1 AIB in the past 3 months; (4) own an iPhone with internet access (due to app requirements of the alcohol sensor); (5) willing to wear the wrist-worn alcohol sensor and activity or sleep sensor ring for 14 days; (6) live or work within a driving distance (~25 miles) of the study site in Tulsa; and (7) fluent in English. The exclusion criteria include (1) currently participating in another study involving sensor wear or EMA and (2) enrolled in high school.

Study staff will respond to emails from interested individuals with contact information for the project coordinator, a brief description of the research, and a secure link to the screening survey. By clicking on the link, participants will be directed to an informed consent statement describing study procedures. Consenting participants will be routed to a screening survey, estimated to take approximately 2 minutes to complete. The screening survey will assess each aspect of the eligibility or exclusion criteria via REDCap [75,76]. Eligible participants will be contacted to schedule the baseline visit via email and will use a Calendly link to schedule their in-person visit. If they are unable to be reached through email, they will be contacted via phone. Participants who do not meet eligibility criteria will be thanked for their time and told they are not eligible.

### Sample Size

We plan to recruit up to 50 participants into this study. Sample size was determined based on feasibility (budget and timeline) related to the pilot funding mechanism. Effect sizes from this pilot will be used to determine appropriate sample sizes and statistical power for future proposals [77].

### Ethical Considerations

Ethics approval was obtained from the institutional review board at the University of Oklahoma Health Sciences (#18534) in August 2025. Survey (implied) informed consent will be obtained prior to completing the screening survey. Written informed consent and HIPAA (Health Insurance Portability and Accountability Act) authorization will be obtained at the beginning of the in-person baseline visit. A Certificate of Confidentiality was obtained from the National Institutes of Health in September 2025. Participants can earn up to US $196 for completing all aspects of the study. Participants will be compensated US $20 for attending the baseline visit, with the opportunity of earning an additional US $20 if they attend the baseline visit within 7 days of being contacted about their eligibility. During the intensive data collection period, participants will earn US $2 per survey (morning and evening scheduled surveys only), equaling up to US $56 for perfect survey completion. Participants can earn an additional US $40 if they complete at least 80% of the daily surveys and wear their sensors at least 80% of the time (verified via temperature and movement sensors; see details about processing below). Participants will earn US $40 for returning their devices and an additional US $20 if they return their devices within 2 business days of completing the intensive data collection period.

This study is considered minimal risk. Psychological risks posed by the research are primarily related to the sensitivity of some of the measures, including thoughts, feelings, and personal difficulties that may be private. It is important to note that the data being collected via surveys or sensors are not diagnostic in nature and will not assess harm to self. Although the alcohol sensor collects (near) real-time measurement of alcohol use, it is not directly translatable to blood alcohol concentration nor immediately available to the study team. Neither survey responses nor sensor data will be monitored in real time. Participants are also asked to report on potentially illegal behaviors, such as drinking under the legal age or using illicit substances. Participants are encouraged to contact the investigators at any time to discuss concerns they might have. Upon request, participants will receive a handout with community resources in the area.

The following steps will be taken to protect individuals’ identities as research participants. First, responses to web-based questionnaires will be identified only by a unique personal identification number, randomly generated for this study. Second, we will keep a master list of names, addresses, emails, phone numbers, and personal identification numbers so that we can compensate participants who complete the study and contact them about their participation. The master list will be stored separately from collected data on a password-protected computer with restricted access or on the university OneDrive. Only approved team members will have access to this list.

### Planned Statistical Analyses: Processing of Intensive Longitudinal Data

#### Social Days

Data reduction strategies will be used so that the primary temporal unit of analysis for this study is the social day. The social day begins with the morning survey time instead of midnight. We will analyze our data using social days because drinking behavior is not encapsulated neatly in midnight-to-midnight “calendar” days. Drinking often either begins in the evening and extends past midnight or begins after midnight, but it is likely experienced as an extension of the previous day.

#### Processing of TAC Sensor Data

We will use previously published (and publicly available [78]) algorithms for processing intensive sensor data [4]. First, we will apply algorithms to filter out observations in which the Skyn device was not worn. These algorithms are based on the Skyn’s temperature and movement sensors. The device will be considered worn if: (1) the recorded temperature is over 28 °Cs, (2) the temperature is over 5° greater than the participant’s minimum recorded temperature during the study period, or (3) the temperature is more than 3° greater than the participant’s minimum recorded temperature and the motion sensor registers motion above 0.01 Gs. Observations indicative of nonwear (low temperature and no movement) will be removed. Second, we will smooth remaining TAC data to remove noise using a 30-minute moving average. The moving average will be centered using data from 15 minutes prior to 15 minutes after to smooth the series. This will result in a variable with a nearly identical mean and reduced variance that is highly correlated with the original. Third, we will bracket data into biological alcohol concentration “episodes”: periods of time in which the TAC remained consecutively above a minimum threshold of 5 μg TAC/L air. New episodes will be marked by 2 TAC values: <5 μg TAC/L air followed by a TAC value ≥5 μg TAC/L air. Episode endings will be marked by a TAC value of 5 μg TAC/L air followed by 2 TAC values <5 μg TAC/L air. Fourth, we will filter out false-positive episodes, characteristically defined by the rates of rise and fall in the TAC that are biologically implausible or impossible. Episodes that are (1) shorter than 45 minutes, (2) shorter than 60 minutes and have a peak ≥400 μg TAC/L air, or (3) have rise or fall rates that do not fall between 20 and 300 will be removed. TAC features will then be extracted for each social day. TAC features include peak TAC, rise rate, rise duration, fall rate, total duration, and area under the curve. If at least 80% of the expected data per social day are present, but the features are missing, it will be assumed that no drinking occurred, and features will be recoded as 0 to represent a nondrinking day. Otherwise, data will be left as missing. Day-level TAC features data will be merged with other day-level data by participant ID and social date.

#### Processing of Oura Ring Sensor Data

Raw data are consolidated via application programming interface and processed using proprietary algorithms. Processed data will be accessed in the Oura Teams environment. The Oura Ring requires at least 3 hours of sleep stage detection during a 24-hour period (between 6 PM and 6 PM) to identify a main sleep cycle. Days without at least 3 hours of sleep stage detection will be considered missing. Potential reasons for missingness may be related to protocol compliance (eg, nonwear or not charging the device) or nights with high levels of movement. Critical components of sleep architecture will be extracted by the social day level using Oura Teams processing pipelines, including sleep latency (minutes to fall asleep), total time and percentage of time in REM sleep, total time and percentage of time in non-REM sleep, total sleep time, total time and percentage of time in deep sleep, and restfulness of sleep. Secondary measures of intoxication (eg, heart rate variability, respiration rate) will be explored. Additional measures related to physical activity (eg, calories, activity level) will also be collected and may be explored in secondary analyses. Day-level sleep data will be merged with other day-level data by participant ID and social date.

### General Modeling Framework

The main outcome of interest in this study is AIBs experienced during the 14-day intensive data collection period. To test our primary hypotheses, each main predictor (combined substance use and sleep) will be examined in separate logistic multi-level structural equation models. Data that are characterized by repeated observations of momentary events will be summarized at the day level as Level 1 (within-person) variables, which will be nested within each participant. Person-level (between-person) differences will be Level 2 variables. Level 1 variables will include the assessments of alcohol (TAC and self-report) and other substance use, sleep, and so forth. Level 2 variables include person-level factors, such as demographics. Our analyses use this nested structure to separate estimates of within-person relationships (day-level) from between-person relationships. Level 1 variables will be person-mean centered, and Level 2 variables will be grand-mean centered. Random paths will be allowed, and Bayesian methods will be used if maximum likelihood models do not converge. Indirect effects (the effects of combined substance use or sleep through alcohol use) will be estimated using either (1) bias-corrected bootstrapped confidence intervals (maximum likelihood) or (2) the posterior distribution of the indirect effect (Bayesian). Models with confidence or credible intervals that do not contain 1 will be considered significant. Covariates will include sex, age, race or ethnicity, and student status.

Pre hoc sensitivity analyses replacing sensor-collected data with self-reported data for alcohol use (eg, drink count) and sleep (eg, sleep quality) will be conducted. For exploratory analyses, moderation will be tested by including interaction terms between Level 1 combined substance use and sleep.

## Results

This study was funded by the Presbyterian Health Foundation in July 2025. Data collection is projected to span January 2026 through June 2026.

## Discussion

### Expected Findings

This study seeks to understand 2 key modifiable behaviors that may be associated with increased AIB risk by leveraging multiple forms of innovative measurement. We anticipate that nights with simultaneous use of alcohol and cannabis, and simultaneous use of alcohol and nicotine, will be associated with increased odds of experiencing an AIB (aim 1). We also anticipate that drinking days preceded by worse sleep will be associated with increased odds of experiencing an AIB (aim 2).

Previous research has begun to explore the impact of other substance use [8,17,39-42] and to a lesser extent, sleep [49], on AIBs and other alcohol-related consequences. This study expands on previous research in a number of ways. First, we will include both college and nonstudent young adults. Nearly all of the research on AIBs has focused on college student samples. According to the alcohol-harm paradox, people with lower socioeconomic status (SES) tend to experience greater alcohol-related harm than those with higher SES, despite often reporting similar (or less) levels of alcohol consumption [79]. Nonstudents are more likely to be of lower SES than those who attend college [80] and may be at increased risk for experiencing alcohol-related harm, in part due to a lack of access to university health resources. Research inclusive of nonstudent young adults is critical to informing alcohol interventions to reduce harm outside of college campuses. Second, we will integrate 2 objective sensors (to measure alcohol use and sleep) with EMA to overcome the limitations of traditional self-report (eg, less reliance on memory). Our approach will allow us to collect objective, fine-grained information on measures of alcohol use (eg, rate of and duration of intoxication) and sleep (eg, REM vs non-REM sleep) otherwise difficult (or impossible) to obtain via self-report. Our use of EMA will also allow us to assess key factors immediately preceding and during a drinking episode. These advanced methodological tools also lend themselves to use in future digital interventions. Each of these tools allows for (near) real-time assessment of our main predictors and outcomes of interest. The use of sensors reduces reliance on self-report, which is frequently inaccurate in inconsistent patterns (ie, heavier levels of drinking often observed in young adults and that are associated with AIBs and examination of measured sleep architecture, including REM sleep), both directly related to the outcome of interest. These methods allow the virtual observation and enhanced understanding of behaviors in real-world contexts.

### Limitations

Despite its many contributions, this study has several limitations. First, the intensive data collection period consists of a single 14-day period. It is possible that this period may not be representative of typical behaviors. Second, only about 50 young adults in a single metropolitan area will be enrolled in the study. Third, it is possible that the alcohol sensors may miss lower-level drinking episodes or the sleep sensors may not be perfectly accurate at differentiating stages of sleep.

### Conclusions

This study will inform efforts aimed at preventing AIBs and reducing alcohol-related harms. The findings from this study will serve as preliminary data on the feasibility and acceptability of the protocol, providing opportunities for conducting future research on a larger scale.

## Supplementary material

10.2196/83980 Peer Review Report 1 Peer review report by the Presbyterian Health Foundation (PHF) Seed Grant Review Committee.

## Abbreviations

AIB: alcohol-induced blackout
EMA: ecological momentary assessment
HIPAA: Health Insurance Portability and Accountability Act
REDCap: Research Electronic Data Capture
REM: rapid eye movement
SES: socioeconomic status
TAC: transdermal alcohol concentration
