# Supplementary material for: Using Alcohol and Sleep Sensors to Understand Blackout Risk in Young Adults’ Natural Settings (The Lights Out Study): Protocol for an Intensive Longitudinal Pilot Study
Source: JMIR Res Protoc. 2026 Jan 21;15:e83980. doi: 10.2196/83980 (PMC12822870; doi:10.2196/83980)
Supplement: Peer Review Report 1 [file resprot-v15-e83980-s001.pdf]

PHF SEED GRANT REVIEW
FACULTY PEER REVIEW CRITIQUE FORM

Requested Start Date:	July 1, 2025
Investigator(s):	Veronica Richards, PhD
Project Title:	Examining the Effects of Polysubstance Use on Blackout Risk in Young Adults' Natural Settings
Requested Budget:	$75,000
Score:	3.48

Critique #1

Overall Impact: This is a well written proposal. The focus on the link between polysubstance use and alcohol induced blackouts is significant and innovative, as most prior research focuses exclusively on single substance use, despite the high co-occurrence of alcohol use (a primary risk factor for AIBs) with cannabis and nicotine. This reviewer was overall enthusiastic about this proposal, though the link with sleep patterns and impact of sleep on AIBs seemed less well-developed or clear. The study proposes to examine poor sleep as both a mediator and a moderator, though this seems to be rather non-specific. It would help if the team could provide a more clear mechanistic link (either mediation or moderation) based on the available literature. The overall core premise of the project is written to examine "temporal patterns of polysubstance use" on increasing acute harm, but sleep is not mentioned, thus sleep feels somewhat secondary to the main focus of the proposal.

1. Significance

Strengths

- The public health impact and consequences of AIBs are clearly laid out

Weaknesses

- The team suggests that sleep is implicated in AIBs, due to loss of REM sleep, though this seems like a rather distal, than proximal risk factor for AIBs. Proximal (same day, episode-level) polysubstance use may be more strongly correlated with AIBs then sleep
- AIBs are stated to occur, in part, because of poor memory consolidation that happens as a result of poor sleep but to fully understand this concept, the team should have included more detail on underlying biological mechanisms that influence AIBs
- Cannabis use and nicotine consumption may also influence sleep, though this is not directly discussed. Some individuals may ingest edible cannabis to help with sleep and cannabis has been shown to improve sleep (though unsure of the impacts on REM sleep). This is also not discussed

2. Investigator(s)

Strengths

- The investigators have the skills and expertise to carry out the proposed project

Weaknesses
- None noted

3. Innovation

Strengths
- The focus on polysubstance use and its impact on AIBs is novel
- Inclusion of TAC sensors, allowing for granular measurement of alcohol consumption is novel

Weaknesses
- None noted

4. Approach:

Strengths
- The measures and the sample seem appropriate to the seed grant and the research questions
- The EMA paradigm is well designed

Weaknesses
- It would help if the team could provide some data on the representativeness of their sample. Are YAs with a history with AIBs who regularly engage in HED representative of YAs who experience AIBs or YAs or who drink more generally?
- Minor weakness: how do the researchers get access to the sleep data from the Oura ring? Does the participant need to return to provide this or is this automatically uploaded/available to the research team by viewing the app?
- The team does not propose an EMA compliance rate. What proportion of the EMAs do they anticipate being completed?
- In addition to random EMAs there are self-initiated EMAs; how are those intended to be used in the analyses? Will random assessments be treated the same in analyses as self-initiated assessments?
- As I noted in the Significance, both cannabis and nicotine can have an impact on sleep. Secondary or exploratory analyses to examine the impacts of other substances on sleep (individually, not with alcohol use) do not seem to be clear.
- In one sentence in the analytic plan, the team states “Moderation will be tested by including and interaction terms between Level polysubstance use and sleep”. There seem to be some typos here so unclear what analysis is proposed here. What does “Level polysubstance use”? Is that number of days engaged in polysubstance use over the entire EMA period?

5. Potential of the Research to Attract Extramural Funding Upon Completion of the Project:

The inclusion of sleep here as a primary mediator or moderator still seems unclear or not entirely compelling, and somewhat disjointed from the rest of the proposal. The team states that the future goal of the proposal is to submit a larger R01 with a longer follow-up to assess trajectories of poly-substance use and sleep over time and a somewhat generic/broad statement about how these targets will be incorporated in a just-in-time adaptive intervention, but without specific details. For

example, if poor sleep predicts subsequent AIBs, OVER AND ABOVE, poly-substance use, would an intervention to improve sleep hygiene (by reducing alcohol use) be warranted? If sleep does not predict AIBs, over and above poly-substance, then a focus on reducing other substance use during drinking episodes would be warranted. The team did not flesh out these potential outcomes, leading to potentially different intervention ideas to pursue in the future. Additionally, the team does not indicate plans for manuscript development/submissions and/or conference abstract submissions to solidify their collaborative working relationship, to help with the success for an R01.

6. Appropriateness of the Application to this Particular Program and Program Guidelines:

This application is appropriate to this particular program and the program guidelines.

7. Additional comments, if any.

N/A

Critique #2

Overall Impact: This proposal aims to examine how temporal patterns of polysubstance use (alcohol, cannabis, and nicotine) influence alcohol-induced blackouts among young adults using wearable sensors and ecological momentary assessment. The proposal addresses a highly significant public health issue and targets a population at high risk for alcohol-related harm. The investigative team is strong with prior experience in related sensing, EMA and alcohol studies. Score-driving strengths include the focus on a clear evidence gap (i.e., timing of substance use in relation to blackout risk), strong preliminary data supporting feasibility of sensor-based measurement and participant recruitment/retention, and an innovative and appropriate study design to meet the study aims. Score-driving weaknesses are concentrated in the Significance and Approach. The proposal lacks a power analysis to justify the sample size, particularly for testing mediation and moderation in Aim 2. The sleep aspects feel tacked on in a superficial way. Recruitment feasibility within the proposed timeframe is not addressed, and no potential problems or alternative strategies are discussed. Cannabis and nicotine are modeled separately, missing the opportunity to examine combined effects that may be central to understanding polysubstance-related AIB risk. The absence of defined feasibility and acceptability measures also limits alignment with the goal of informing a future R01. These issues only somewhat diminish enthusiasm for an otherwise outstanding application.

1. Significance

Strengths

- Characterizing patterns of polysubstance use that increase risk for alcohol-induced blackouts (AIBs) among young adults (YAs) is important work. YAs have high rates of heavy drinking, AIBs, and polysubstance use.
- The proposed research addresses important limitations in evidence (i.e., timing of other substance use relative to alcohol use is unknown).

Weaknesses

- The inclusion of sleep feels loosely connected to the central premise of the proposal, and the role of sleep as a mediator or moderator in the polysubstance–AIB link is not clearly justified or well-integrated into the overall scientific rationale.

2. Investigator(s)

Strengths

- The study team has deep expertise in wearable sensors, ecological momentary assessment (EMA), recruitment and retention of YAs who engage in heavy drinking, and intensive longitudinal methods.

Weaknesses

- Team expertise is primarily in alcohol. Little expertise with polysubstance use on the team.

3. Innovation

Strengths

- Innovative use of sensors to measure alcohol use, AIBs, and sleep.
- The study design addresses key limitations in existing polysubstance use research (e.g., timing of use), even if not explicitly stated.

Weaknesses

- None noted.

4. Approach:

Strengths

- Strong preliminary data from four prior studies demonstrating the team's expertise in wearable sensors, EMA, recruitment and retention of feasibility, and statistical methods.
- The proposal incorporates near real-time measurement of alcohol use, AIBs, and sleep.
- Robust plan for using multiple methods to recruit participants.
- Appropriate statistical analysis plan for the proposed aims.

Weaknesses

- The recruitment, screening, and baseline visit aspects of the proposal are underdeveloped, as are potential problems and alternative strategies.
- While the application presents strong preliminary data demonstrating experience with measurement and recruitment approaches, it does not address the feasibility of recruiting the target sample within the proposed timeframe. This omission makes it difficult to assess whether recruitment goals are realistic and achievable.
- The application states that the study will inform a future R01 by providing feasibility and acceptability data, but no specific measures are included. Adding an acceptability measure to the end-of-study EMA would be valuable.
- No **power calculations** to justify the adequacy of the sample for testing the stated aims are provided. The power analysis should also speak to being adequately powered to the specified mediators and moderators (e.g., sleep).
- Analyzing cannabis and nicotine separately may miss how their combined use contributes to AIB risk.

5. Potential of the Research to Attract Extramural Funding Upon Completion of the Project:

- Strong likelihood to attract extramural funding, with a clear next step to obtain a NIH R01 grant.

6. Appropriateness of the Application to this Particular Program and Program Guidelines:

- Strong feasibility and scientific merit.
- Rigorous study design and measurement.
- Data analysis is appropriate.
- Strong preliminary data and important project, with strong potential to attract extramural funding upon completion.
- PI within 5 years of full academic appointment.
- Strong track record of success by investigative team related to concepts in the proposed project.

7. Additional comments, if any.

- None noted.
